# Multiple Two-Way Time Message Exchange (TTME) Time Synchronization for Bridge Monitoring Wireless Sensor Networks

**DOI:** 10.3390/s17051027

**Published:** 2017-05-04

**Authors:** Fanrong Shi, Xianguo Tuo, Simon X. Yang, Huailiang Li, Rui Shi

**Affiliations:** 1 Robot Technology Used for Special Environment Key Laboratory of Sichuan Province, Southwest University of Science and Technology, Mianyang 621010, China; li-huai-liang@163.com; 2 College of Chemistry and Environment, Sichuan University of Science and Engineering, Zigong 643000, China; 3 Advanced Robotics and Intelligent Systems (ARIS) Laboratory, School of Engineering, University of Guelph, Guelph, ON N1G 2W1, Canada; syang@uoguelph.ca; 4 State Key Laboratory of Geohazard Prevention and Geoenvironment Protection, Chengdu University of Technology, Chengdu 610059, China; shirui0601@126.com

**Keywords:** wireless sensor networks, bridge monitoring, time synchronization, time message exchange, timeout constraint, maximum likelihood estimation

## Abstract

Wireless sensor networks (WSNs) have been widely used to collect valuable information in Structural Health Monitoring (SHM) of bridges, using various sensors, such as temperature, vibration and strain sensors. Since multiple sensors are distributed on the bridge, accurate time synchronization is very important for multi-sensor data fusion and information processing. Based on shape of the bridge, a spanning tree is employed to build linear topology WSNs and achieve time synchronization in this paper. Two-way time message exchange (TTME) and maximum likelihood estimation (MLE) are employed for clock offset estimation. Multiple TTMEs are proposed to obtain a subset of TTME observations. The time out restriction and retry mechanism are employed to avoid the estimation errors that are caused by continuous clock offset and software latencies. The simulation results show that the proposed algorithm could avoid the estimation errors caused by clock drift and minimize the estimation error due to the large random variable delay jitter. The proposed algorithm is an accurate and low complexity time synchronization algorithm for bridge health monitoring.

## 1. Introduction

Bridges are an important part of transportation systems, Structural Health Monitoring (SHM) of bridges is indispensable and multiple sensors such as acceleration, displacement, temperature and strain sensors [1,2,3,4,5,6] are employed to collect the real-time information about bridges. As described in [1,5,6] synchronized sensing is necessary to ensure accuracy in SHM applications, so time synchronization is a key technology in the wireless sensor networks (WSNs) used for SHM of bridges and is important for collaborative tasks, intelligent sleeping and data consistency [5,6,7,8]. WSN nodes use local hardware clock sources to set up their local times, which differ from each other. There are two main reasons for these differences. The nodes are initialized at different moments so there is clock offset among nodes. More than that, the clock sources have variable clock frequency offsets which are due to the clock manufacturing techniques used and the changing environments. Hence in networks there is clock skew and variable clock offsets. Time synchronization algorithms are proposed to correct the local time information and force the time notions of different nodes to be consistent. It has been used in many WSN applications, such as location [9,10,11,12], environmental monitoring [13,14,15,16], data acquisition [17,18,19], delay measurement [20], power saving [21] and so on.

Accurate clock offset estimation is one of the key factors to improve the time synchronization. One type of proposed time synchronization algorithms correct the clock offset between nodes [13,22,23]. These approaches provide for simple calculations and cost less to achieve, but because of the influence of the ignored clock frequency offset, these time synchronization algorithms need re-synchronization to maintain the precision. Some improved algorithms introduced joint skew-offset correction [9,22,23,24,25,26,27,28,29,30,31]. Additionally, these algorithms correct the relative clock speeds and reduce the clock skew, so the time synchronization algorithms with joint skew-offset correction are better for synchronization over a long time. Actually, almost all of the joint clock skew-offset correction time synchronization algorithms use the existing clock offset estimations to calculate the relative clock speed and compensate the clock skew. Obviously, accurate clock offset estimations are important for the clock skew estimate of time synchronization algorithms.

The early time synchronization algorithms such as RBS [21] use a large number of beacon broadcasts to synchronize a pair of receivers. The receivers record the arrival time of the beacon signals and exchange it among each other, then the clock offset and clock skew can be calculated. Absent MAC-layer timestamps, RBS provides a rough time synchronization. The improved CESP algorithm [23] introduces the reference node MAC-layer time stamp into the beacons’ broadcasts. It uses synchronization coefficient exchange instead of time information exchange and the communication overhead is reduced. TPSN [24] is a time synchronization algorithm for sender to receiver and uses rough time stamps, but its clock offset estimate error is nearly two times smaller than that of RBS.

FTSP [9] floods the reference time information to the networks and employs multiple MAC-layer time stamps to improve the time stamp precision. The flooding packets, which load the send time stamps of reference nodes, are broadcasted periodically. The receivers estimate the clock offset and skew by exchanging their time stamps directly. PulseSyn [25] is a rapid-flooding time synchronization algorithm that believes that all of the nodes should to broadcast the reference time information as soon as possible. It aims to solve the problem of the amplified error caused by the delay before a node broadcast gets its received reference time information. FCSA [26] verifies that there is a smaller time synchronization error if the nodes have less clock frequency differences. It uses a clock speed agreement algorithm to improve the slow-flooding time synchronization algorithm in FTSP. There are many other algorithms which are complete distributed time synchronization algorithms. GTSP [27] and EGSync [28] were proposed to minimize the skew of any two neighborhood nodes. The consensus-based algorithms ATS [29], MTS [30] and CCS [31] are effective for dynamic topology networks. These algorithms have slow convergence rates for accurate time synchronization. SHM of bridges is a special application of WSNs, where the traditional synchronization methods may not meet its needs and many time synchronization algorithms are proposed for the data gathering and data fusion [7,8,32,33]. Xiao et al. [32] and Hu et al. [7] focused on the energy efficiency of time synchronization, and improved the previous algorithms and proposed an energy balanced time synchronization algorithm to lengthen the whole WSNs’ life. Xu et al. [8] proposed an asymmetrical clock synchronization algorithm for bridge health monitoring WSNs to monitor real-time bridge conditions. To improve the TPSN [24] for ensuring the synchronization accuracy without consuming more power, Gong et al. [33] proposed a partial TPSN time offset synchronization scheme for bridge health diagnosis WSNs.

The transmission delay results in a large error for clock offset estimation, especially for the random variable delay portion. Specific time message exchange mechanisms are employed by time synchronization algorithms to reduce these disadvantages. WSNs are a complete distributed system. The hardware resources and computational power are limited. The clock frequency of a node could not be measured directly. Its transmission delay is unknown and variable, so the time synchronization algorithms always estimate the clock offset from time information transmissions [34,35]. One-way broadcast time messages are employed for synchronizing receiver to receiver [25,29]. The reference nodes broadcast their time information packets periodically and the receivers use the time message to correct their local times. This mechanism removes the variable delay from clock offset estimate at the sender but a variable delay is introduced into the estimation directly at the receiver.

The two-way message exchange (TTME) clock offset estimator was first proposed in TPSN [24] for wireless sensor network time synchronization. It uses pairs of transmissions that are named uplink and downlink to make four time stamps. Then the time stamps are used to estimate and correct the clock offset among nodes. Chaudhari et al. [36] and Wang et al. [37] employed an ACK mechanism TTME, but not a dedicated time message packet. These protocols only use the uplink time stamp for clock offset estimation. Jeske [38] proved that the MLE exists for TTME. The transmit delays were denoted as fixed portions and variable portions as decomposed by Abdel-Ghaffar [39]. To calculate the clock offset of MLE, it is assumed that there are a subset of TTME observations which have same fixed clock offset. In employing the minimum observations of uplink and downlink the clock offset of MLE can be calculated. Noh et al. [40] assumed that the probability distribution function of random variable delay was either an exponentially distribution function or a Gaussian distribution function. They proved the Cramer-Rao lower bounds (CRLBs) for the clock offset MLE under these different delay models.

However, a time synchronization scheme for WSNs in bridge health monitoring that has remarkably low complexity and ease of implementation still needs to be developed. In this paper we focus on MLE of two-way message exchange (TTME) clock offset estimation. For MLE of clock offset estimate, a sufficiently large subset of TTME observations is a prerequisite. In addition, a same fixed clock offset for the subset is beneficial for accurate clock offset estimation.

TTME like TPSN may not meet the MLE of clock offset estimation. To simplify the description, the TTME similar to TPSN is named traditional TTME. The traditional TTME ignores the random variable delay and introduces it into the clock offset estimation. Additionally, the clock offset due to the clock drift is not considered. Since the assumption that nodes have fixed clock offset is not always valid and the TTME in TPSN is an ideal clock offset estimate model, the main clock offset estimate error for these algorithms are caused by random variable delay and clock drift. Especially when there are large transmission delay jitters and large software blocking latencies, the estimate errors will increase predictably.

Our work aims to meet these requirements. The multiple TTMEs in this paper provide an effective clock offset MLE approach for time synchronization in WSNs which is a potential application for SHM of bridges. The proposed algorithm demands less calculation and is easy to embed in the WSN application. This paper takes an optimized TTME approach to achieve high precision time synchronization for the data acquisition in bridge health monitoring WSNs. We have discussed the details of the clock offset continuously increasing, then optimized the execution time and speed of the TTME to meet the assumption in TPSN. The maximum likelihood estimation (MLE) is employed to reduce the clock offset estimate errors due to the random variable delay. Then the multiple TTMEs algorithm aims to provide as many TTME observations as possible for MLE. The multiple TTMEs is different from the traditional TTME. It employs the timeout constraint to avoid the clock offset estimation errors due to the uncertain communication links and response delays caused by the task blocking latencies of software. Compared to TPSN, the proposed algorithm obtains more accurate clock offset estimations and lower clock skews.

The rest of this paper is organized as follows: in Section 2, we introduce the system model. In Section 3, we analyze the details of the continuously increasing clock offset, and derive the restraints for the fixed clock offset TTME. The multiple TTMEs algorithm is proposed in Section 4. The clock offset and clock skew estimate are described in Section 5. The simulation results are shown in Section 6 and finally, we present our conclusions in Section 7.

## 2. System Models

In this section, we introduce the system models that are used in our work. The bridge monitoring WSN model is defined as a graph, G=(V,E). The subset of nodes is defined as V={1,…,n}. The bidirectional communication link (edge) subset is defined as E⊆(V×V). An arbitrary node i has a neighborhood nodes subset $N_{i}=\{i,j\}\in E$ and node i can only communicate with the nodes in $N_{i}$. The distance between arbitrary nodes {i,j}∈V is defined as the number of edges on the shortest path between the two nodes. The *diameter* is defined as the maximum *distance* of any two nodes in the graph G.

### 2.1. Time Synchronization for SHM of Bridges

Based on the shape of bridges, the topology of bridge monitoring WSNs can be designed as a spanning tree [1], so the time synchronization algorithm employs the tree to maintain the time synchronization. The MAC layer time-stamping technology is employed to make time stamps. Root is the reference node and starts the TTME to force its child nodes to synchronize to itself firstly. Then the synchronized nodes become reference nodes and start the new TTME to synchronize their child nodes until the network has synchronized the end node along the spanning tree.

**Figure 1:**
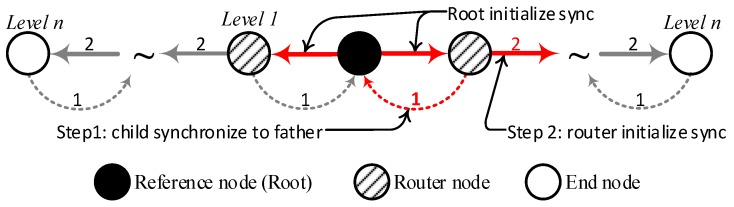
The architecture of time synchronization for SHM of bridges.

The architecture of time synchronization for SHM of bridges is shown in Figure 1. It assumes that a spanning tree is created. The root of the tree is the reference node of the network, which employs GPS as an external clock source. The routers are forwarding nodes for the time synchronization. The high level nodes always employ their parent as reference node and synchronize to it. After the spanning tree is created, the root initializes the time synchronization by an initialization broadcast. The router node, which has received this broadcast, is synchronized to the root, and then sets itself up as a reference for its own nodes. The router uses two steps to flood the time synchronization through the network: (1) it requests a synchronization response from the reference node to estimate the clock offset and clock skew; (2) it compensates the local clock source parameter, and then transforms itself into the reference node of its son nodes and initializes the synchronization.

### 2.2. Clock Model

The bridge monitoring WSN nodes are driven by hardware clock sources which usually are crystal oscillators. The time notion is created by counting the hardware clock pulses. The clock speed of the node i is the increment of its time count in the specified time range, i.e., $h_{i}\left(\tau \right)$. $C_{i}(t)$ is the time counting of node i at time t, and $C_{i}(t)$ is defined as:

(1) $$C_{i}\left(t\right)=\int_{0}^{t}h_{i}\left(\tau \right)d\tau$$

If f is the nominal frequency of hardware clock in node i, then the granularity of the time counter is δ=1/f. The time notion of the node i can be defined as:

(2) $$L_{i}\left(t\right)=\delta C_{i}\left(t\right)+x_{i}(0)$$

$L_{i}(t)$ is the logic time of node i at time t. $x_{i}(0)$ is the logic time of node i at time 0 and it is the initial time of node i. If t=0 is the moment that the nodes synchronize to each other, then the relative time model and clock offset model of pairs nodes i and j are defined as:

(3) $$L_{j}\left(t\right)=L_{i}\left(t\right)+O_{ij}\left(t\right),$$

(4) $$O_{ij}\left(t\right)=\delta (C_{j}\left(t\right)-C_{i}\left(t\right))+x_{j}(0)-x_{i}(0),$$

where $L_{i}(t)$ and $L_{j}(t)$ are logic time of i and j at time t. $O_{ij}(t)$ is the clock offset between i and j at time t. The $O_{ij}\left(0\right)$ is defined as the time synchronization error.

For the arbitrary node i∈V and arbitrary node $j\in N_{i}$, the *local skew* is defined as the maximum relative clock offset $O_{ij}(t)$ between nodes i and j at time t, i.e., $Maximum_{i\in V,j\in N_{i}}\{O_{ij}(t)\}$. For the arbitrary node i,j∈V, the *global skew* is the maximum relative clock offset $O_{ij}(t)$ between nodes i and j at time t, i.e., $Maximum_{i\in V,j\in V}\{O_{ij}(t)\}$. The time synchronization algorithm is employed to enforce $O_{ij}(t)\to 0$.

### 2.3. TTME Clock Offset Estimation Model

**Figure 2:**
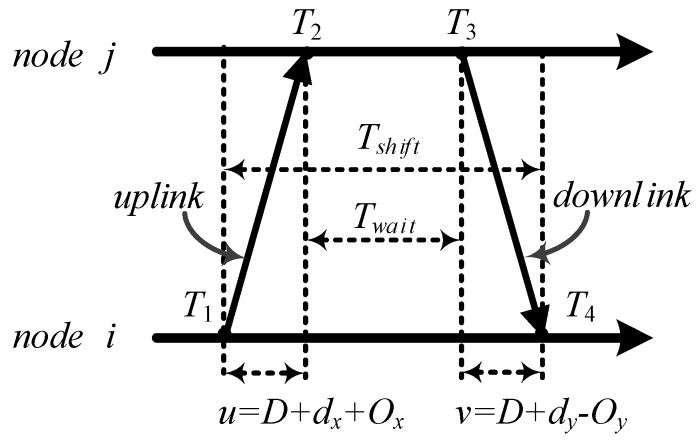
Two-way time message exchange between nodes i and j; there are reasonable different clock offsets caused by clock frequency, as $O_{x}$ for uplink and $O_{y}$ for downlinks.

To simplify the description, j is defined as the reference node and i is defined as an asynchronous node. The details of traditional TTME are shown in Figure 2. The node i needs to be synchronized. j is the reference node. The TTME has two steps. In the first step, i sends a short message to j. This message is just filled with the identity number of nodes i and j. Once the message is sent successfully, i will create a local logic time stamp $T_{1}$. The node j will make the local logic time stamp $T_{2}$ after the message is received. This step aims to create time stamps $T_{1}$ and $T_{2}$ and is defined as uplink. The second step is downlink. The reference node j respond with the TTME at $T_{3}$. j sends another short message which contains the time stamps $T_{2}$ and $T_{3}$ to i. Node i records the time of the message arrival as time stamp $T_{4}$. This step aims to create time stamps $T_{3}$ and $T_{4}$. u is the duration time for TTME *uplink*, i.e., $u≜T_{2}-T_{1}=D+d_{x}+O_{x}$. v is the duration time for TTME *uplink*, i.e., $v≜T_{4}-T_{3}=D+d_{y}-O_{y}$. D is defined as the fixed transmission delay between i and j. $d_{x}$ and $d_{y}$ are the variable transmission delay. $O_{x}$ and $O_{y}$ are the clock offset of i relative to j.

$T_{shift}$ is the time interval between $T_{1}$ and $T_{4}$ on node i. It is the duration time of TTME. $T_{shift}≜T_{4}-T_{1}$. The $T_{wait}$ is the time interval between $T_{2}$ and $T_{3}$ on node j. It is the response delay of TTME. $T_{wait}≜T_{3}-T_{2}$. $T_{wait}$ is a variable latency from software delay, such as the interrupt response delay, priority queuing delay, software blocking latencies. Furthermore, $T_{shift}=T_{wait}+u+v$. $d_{x}$ and $d_{y}$ are described as the random variable with exponential distribution. For this reason we assume that $T_{shift}$ is random and variable with exponential distribution, and the mean is unknown.

Traditional TTME clock offset estimation is given by Equation (5). There is clock offset $O_{ij}$ for the TTME uplink and downlink among nodes ascribed to the clock frequency offset. $O_{y}≜O_{x}+O_{ij}$. $O_{x}$ and $O_{y}$ are relative clock offset for node i during TTME.

(5) $${\hat{O}}_{x}=\frac{\left(T_{2}-T_{1}\right)-(T_{4}-T_{3)}}{2},$$

Equation (5) can be rewritten as:

(6) $$O_{x}={\hat{O}}_{x}+\frac{\left(d_{x}-d_{y}\right)}{2}+\frac{O_{ij}}{2},$$

where $O_{x}$ is the clock offset at the beginning of TTME between node i and j. ${\hat{O}}_{x}$ is the estimation for $O_{x}$. TTME time synchronization uses ${\hat{O}}_{x}$ to correct the local logic time instead of $O_{x}$. Equation (6) shows that estimate errors are mainly caused by $d_{x}$, $d_{y}$ and $O_{ij}$.

The TTME uses time stamps to estimate the clock offset, so it is necessary to discuss the time stamping method. For different approaches, there are differences in the precision of time stamps. RBS creates the time stamp at the application layer and an uncertainty delay at the sender is introduced into the time stamp. TPSN creates its time stamping at the MAC-layer when the packet is about to be transmitted. This reduces the error caused by any uncertainty delay. FTSP uses multiple MAC-layer time stamps which were created by bytes transmission and alignment to reduce the jitter of time stamping. The multiple MAC-layer time stamps approach can obtain a higher precision time stamp so it is also employed by this paper.

## 3. Preliminaries for TTME Clock Offset Estimate

The proposed algorithm has proven that $d_{x}$ is not equal to $d_{y}$ and special probability distribution functions are employed to estimate them [39]. Additionally,$\;d_{x}$ is not equal to $d_{y}$ and $O_{ij}$ is not equal to zero. In this Section, we analyze the details of clock offset changing and then the multiple TTMEs method is proposed to create the subset of fixed clock offset observations for MLE.

### 3.1. Increasing Clock Offset

The clock source of bridge monitoring nodes is usually driven by a rough crystals oscillator. The max frequency offset $a_{max}$ could be up to dozens of ppm or even hundreds of ppm. If the clock frequency offset is relatively stable in a short time then the clock offset increase speed is fixed, the max clock offset can be calculated.

Assume that the frequency of nominal clock source r is f. The frequency offset of the arbitrary clock source i is $a_{i}$ ppm. $\beta =10^{6}$. The frequency of clock source i can be rewritten as $f_{i}$ in Equation (7). Both $a_{i}$ and $f_{i}$ are unknown:

(7) $$f_{i}=f(1+\alpha_{i}/\beta )$$

(8) $$T_{tick}=1/f$$

$T_{tick}$ in Equation (8) is the timing granularity of node i. It is the ideal clock period for a logic time counter but not the real period of clock i. When set the angular rate $w_{i}=2\pi f_{i}$, the real time phase is $\varphi_{i}\left(t\right)=w_{i}t$. The real time phase difference $\varphi_{r}(t)$ between i and r can be written as:

(9) $$\varphi_{r}\left(t\right)=\varphi_{f}\left(t\right)-\varphi_{i}\left(t\right)=2\pi \alpha_{i}ft/\beta$$

(10) $$\gamma_{r}\left(t\right)=\varphi_{r}\left(t\right)/2\pi =\alpha_{i}ft/\beta$$

(11) $$O_{r}\left(t\right)=\gamma_{r}\left(t\right)T_{tick}=\alpha_{i}t/\beta$$

where $\varphi_{f}(t)$ is the phase of r at t. $\varphi_{i}(t)$ is the phase of i at t.$\;\gamma_{r}(t)$ in Equation (10) is the counts of differences for i compared to r at time *t*. The $O_{r}(t)$ in Equation (11) is the relative clock offset model of node i. The reference clock is r. $O_{r}(t)$ is a continuous clock offset for the hardware clock.

In short, the clock offset $O_{r}(t)$ is increasing continuously. $1+a_{i}/\beta$ is the increasing speed of clock i and is due to the frequency offset, so there is variable clock offset in TTME. The estimate errors of clock offset ${\hat{O}}_{x}$ in Equation (6) are unavoidable.

### 3.2. Fixed Clock Offset for Logic Time

The fixed clock offset does not exist in the hardware clock, but in logic time notion. Nodes employ a counter or timer to set up the logic time. This time notion is not a continuous time but a discrete time which increases by an integer multiple time of granularity $T_{tick}$. We define $L_{i}$ as the logic time of node i. $N_{i}$ is the clock pulse count of i at t. $T_{i}$ is the actual period of clock i. Then we can write $L_{i}\left(t\right)$ as:

(12) $$L_{i}\left(t\right)=N_{i}T_{tick}$$

(13) $$N_{i}=\lfloor t/T_{i}\rfloor =\lfloor (1+\alpha_{i}/\beta )ft\rfloor$$

The time granularity of logic time $L_{i}$ is $T_{tick}$, but not the period $T_{i}=1/f_{i}$ and $T_{tick}\neq T_{i}$. $N_{i}$ is rounded down $t/T_{i}$. The logic time $L_{i}$ is a discrete representation of the real time t. By rewriting Equations (10) and (11), we have the logic time offset of nodes i and j given as:

(14) $$\gamma_{L}\left(t\right)=\lfloor \alpha_{i}ft/\beta \rfloor$$

(15) $$O_{L}\left(t\right)=\;L_{i}\left(t\right)-\;L_{j}\left(t\right)=T_{tick}\gamma_{L}\left(t\right)$$

where $\gamma_{L}\left(t\right)\in N$ is the number of cycles difference between nodes. $O_{L}(t)$ is the logic time offset of nodes i and j. The logic time offset is different from hardware clock offsets. As Equations (14) and (15) show, t and $a_{i}$ are variable. For the fixed t, the larger clock frequency offset leads to larger clock offset. If $\alpha_{i}<\alpha_{j}$, in a bounded time $t_{fixed}$:

(16) $$\underset{a_{i}\to a_{max}}{\lim}O_{L}\left(t_{fixed}\right)=T_{tick}a_{max}ft_{fixed}/\beta$$

(17) $$\underset{a_{i}\to 0}{\lim}O_{L}\left(t_{fixed}\right)=0$$

**Assumption** **1.** If the increment of $O_{L}(t)$ is not larger than 1 $T_{tick}$, then there is a fixed logic clock offset $O_{L}(t)$.

**Figure 3:**
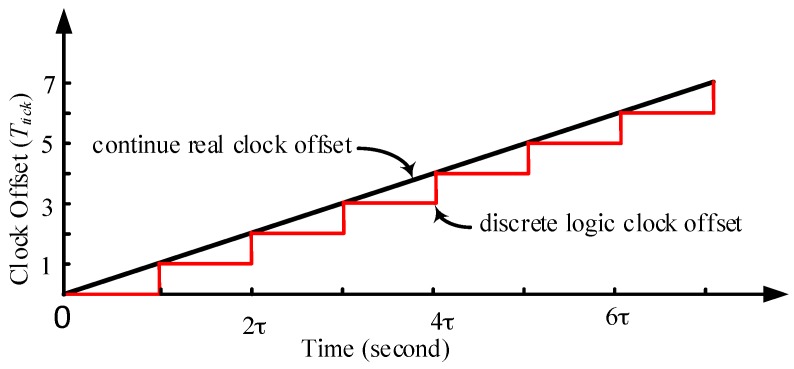
The hardware clock offset and logic clock offset. Assume there is fixed clock frequency offset, the real clock offset $O_{r}(t)$ is linear and continually increases. The logic clock offset is discrete, since the logic clock is driven by the clock granularity $T_{tick}$, the logic clock offset is the integer multiple of $T_{tick}$. The term τ is the cost of the clock offset increment in one $T_{tick}$, set γ(t)=1 in Equation (10), then $\tau =t=\beta /\alpha_{max}f$.

The logic time offset $O_{L}(t)$ is gradually increasing as shown in Figure 3. In the period that $\gamma_{L}(t)$ is non-integer, $O_{L}(t)$ is a fixed value. The TTME clock offset is $O_{L}(t)$ but not $\;O_{r}\left(t\right).$

Therefore the fixed clock offset for TTME exists with restrictions. As Figure 3 shows, the logic time offset is a discrete integer multiple time granularity so that at the range of nτ and (n−1)τ ($n\in N_{+}$), there is a fixed logic clock offset. When there is phase offset among the clock sources, the valid pulse edge for different clock sources arrives at the time counter alternately, so there is an unavoidable clock offset of less than $T_{tick}$. If the valid pulse edge of i arrives ahead and the clock offset is $\alpha T_{tick}(\alpha <1)$, the clock offset is $(1-\alpha )T_{tick}$ when the valid pulse edge of i arrives. This will reduce the precision of clock offset estimation and intensify the clock skew.

## 4. The Multiple TTMEs for Time Synchronization

Based on the above analysis the estimate error of Equation (6) is given by $O_{ij}$. The define the time stamp $T_{1}$ of TTME as the time origin, set t=0, while for the logic time notion the clock offset increment is given by $O_{L}$ but not $O_{r}$, so $O_{ij}=O_{L}$. Based on Assumption 1 the clock offset model is given by:

(18) $$O_{y}=\{\begin{array}{ll} O_{x} & t\leq T_{o}\\ O_{x}+O_{L}\left(t\right) & t>T_{o} \end{array}$$

where $T_{o}$ is the time cost that the clock offset increment grows from zero to one $T_{tick}$. As Equations (14), (15) and (18) show, for a shorter τ, there is the greater probability that $O_{x}$ equal to $O_{y}$ in Equation (13) and the smaller estimate error $O_{ij}$ for ${\hat{O}}_{x}$. So if $T_{shift}<T_{o}$, $O_{y}=O_{x}$. Set $\gamma_{L}\left(t\right)=1$, $T_{o}=t$ and $O_{L}\left(t\right)=T_{tick}$, Equation (15) can be rewritten as:

(19) $$T_{o}<\beta /\alpha_{max}f$$

where $T_{o}$ in Equation (19) will satisfy Equation (18). If the cost time of TTME is less than $T_{o}$, then its observations has a fixed clock offset. $a_{ij}<\alpha_{max}$ is the relative frequency offset between i and j.

The multiple TTMEs are proposed based on TTME. Firstly, we need to calculate the $T_{offset}$ which is the time cost for the increase in one$\;T_{tick}$. If $O_{ij}=T_{tick}$, $t=T_{offset}$ and$\;O_{r}\left(t\right)=T_{tick}$, Equation (11) can be rewritten as:

(20) $$T_{offset}=\beta T_{tick}/\alpha_{max}$$

For a known $T_{tick}$, $T_{offset}$ is determined by $\alpha_{max}$. As shown by Equation (18), the time cost of the local clock offset increment is smaller than one $T_{tick}$ when the time cost is smaller than $T_{offset}$ in Equation (20), so the TTME observations at the period of $T_{offset}$ have the same fixed clock offset which is defined as $O_{a}$. 

The multiple TTMEs algorithm is proposed to establish n times TTME at the period $T_{offset}$ and obtain the TTME observations set ${\left\{\left(U_{k},\;V_{k}\right)\right\}}_{k=1}^{n}$. $d_{k,x}$ is the variable delay of uplink for the TTME k. $d_{k,y}$ is fixed delay of downlink for the TTME k. $T_{k,1}\sim T_{k,4}$ is the time stamps for the TTME k. $T_{shift,N}\;$ is the time cost for the N times TTME, $T_{shift\;,N}=(T_{N,4}-T_{1,1})$. N is the expectation of multiple times for multiple TTMEs, i.e., the number of observations (Algorithm 1, line 2). It is important parameter for multiple TTMEs time synchronization algorithm and should be set as constant. The multiple TTMEs are defined as Algorithm 1.

**Algorithm 1.** Multiple TTMEs with timeout constraint■ ***Initialization*** ***1.***
*set*
$T_{tick}\beta /a_{max}\to T_{offset}$*.* ***2.***
*set*
$\rho T_{offset}\to T_{limit}$
*,*
num=1*.*
■ ***TTME***
 ***3.***
*run the Two-way message exchange.*
 ***4.***
$U_{num}=T_{num,2}-T_{num,1}$*.*
 ***5.***
$V_{num}=T_{num,4}-T_{num,3}$*.*
■ ***Timeout detection***
 ***6.***
$T_{shift,N}\leftarrow (T_{num,4}-T_{1,1})$
*,*
num=num+1*.*
 ***7.***
*if*
$T_{shift,N}<\;T_{offset}$
*then jump to 3.*
 ***8.***
$N=num,\;save\;{\left\{\left(U_{k},\;V_{k}\right)\right\}}_{k=1}^{N}$*.*


**Figure 4:**
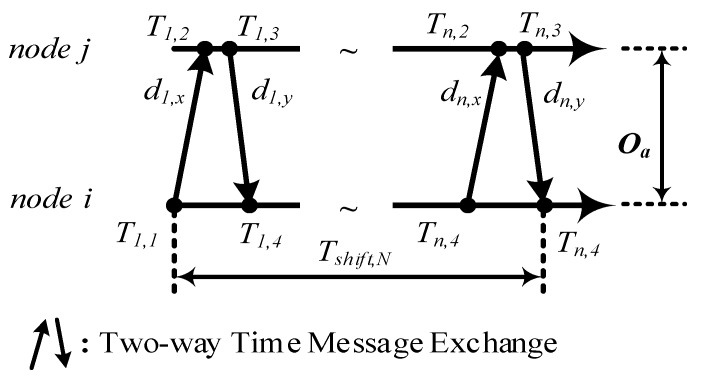
Multiple TTMEs . The TTME is repeated for n rounds under the restriction that it should continue until $T_{shift,N}<T_{offset}$. The TTME observations have a same fixed clock offset $O_{a}$.

Multiple TTMEs employ $T_{offset}$ as time out restraint (Algorithm 1, line 1). $T_{offset}$ determines the times of TTME. The $T_{limit}$ is $\rho T_{offset}$ and 0≤ρ≤1/N. The temporary variable num is used to record the number of valid observations. TTME is repeated until there is timeout (Algorithm 1, line 3–6). Timeout detection is employed to guarantee the TTME observations to have the same fixed clock offset (Algorithm 1, line 6,7). The details of the multiple TTMEs protocol are shown in Figure 4. The observations $U_{k}≜T_{k,2}-T_{k,1}$, $V_{k}≜T_{k,4}-T_{k,3}$. The fixed clock offset $O_{a}$ is:

(21) $$O_{a}=\frac{U_{k}-V_{k}}{2}-\frac{d_{k,x}-d_{k,y}}{2}.$$

The estimation k for $O_{a}$ is ${\hat{O}}_{a,k}$, k∈(1,n), Equation (21) is rewritten as:

(22) $${\hat{O}}_{a,k}=\frac{U_{k}-V_{k}}{2}=O_{a}+\frac{d_{k,x}-d_{k,y}}{2}.$$

Compared to Equations (6) and (15), the observations satisfy Equation (22) when $T_{shift}<T_{o}$. According to Equation (19), there is a larger $T_{o}$ for the smaller clock frequency offset a. Then there is a larger expectation for the multiple TTMEs number N $T_{shift,N}$ so that it is more reliable to estimate the delay with a distribution function. The multiple TTMEs guarantee a same fixed clock offset for the time stamps and aim to obtain an observation set as large as possible. The clock offset estimate error for ${\hat{O}}_{a,k}$ is mainly due to the random variable delay $d_{x}$ and $d_{y}$.

The constant ρ needs to be set at an appropriate value. A smaller ρ leads to a smaller $T_{limit}$ and a tighter restriction for timeouts. There will be a smaller $T_{shift,N}$ for a reliable TTME so that the TTME has greater probability of getting a fixed clock offset for time stamps and higher precision of clock offset estimation for time synchronization. Conversely, a larger ρ means that there is a loose constraint for TTME. The probability for the fixed clock offset time stamps is smaller. There will be rough clock offset estimation for TTME. In addition, ρ does not only determine the precision of clock offset estimation, but also relates to the efficiency of time synchronization. A tight timeout restriction leads to a greater probability of TTME retries (Algorithm 1, line 7), and can even lead the TTME into an infinite loop (Algorithm 1, line 7), so a maximum retry number should be employed to avoid this worse case. Therefore, an exact ρ will balance the accuracy and convergence speed of time synchronization algorithm. A big $T_{shift,N}$ makes the TTME of Equation (5) invalid. The multiple TTMEs hold the $T_{shift,N}$ and avoids the additional error $O_{ij}$.

## 5. The Clock Offset and Clock Speed Correction

The multiple TTMEs aim to correct the relative clock offset and clock speed. The clock offset estimation refers to the precision of time synchronization, while the clock speed estimation refers to the clock skew for networks.

${\hat{O}}_{ij}(\varepsilon )$ is defined as the ε time relative clock offset estimation at node i. $h_{i}^{j}\left(t\right)$ is defined as the relative clock speed offset at t for node i. ${\hat{h}}_{i}^{j}\left(t\right)$ is the estimation for $h_{i}^{j}\left(t\right)$. The root node r has $h_{r}^{r}\left(t\right)={\hat{h}}_{r}^{r}\left(t\right)=1$.

**Algorithm 2.** MLE clock offset and linear regression clock speed, if I is not root, j is reference node for i.■ ***Initialization*** ***1.***
*set*
ε=1*.*
 ***2.***
*set*
${\hat{h}}_{i}^{j}\left(t\right)=1$*,*
$M\in N_{+}$*.*
 ***3.***
*receive and save*
${\hat{h}}_{j}^{r}\left(t\right)$*.*
■ ***Clock offset MLE*** ***4.***
*load*
${\left\{\left(U_{k},\;V_{k}\right)\right\}}_{k=1}^{n}$*.*
 ***5.***
${\left\{\left(U_{k},\;V_{k}\right)\right\}}_{k=1}^{n}\;\to \;{\hat{O}}_{ij}(\varepsilon )$*.*
 ***6.***
$save\;{\hat{O}}_{ij}\left(\varepsilon \right),\;\varepsilon =\varepsilon +1$*.*
■ ***Linear regression clock speed***
 ***7.***
*if*
ε<M
*then then jump to 12.*
 ***8.***
*else load*
${\left\{{\hat{O}}_{ij}(k)\right\}}_{k=\varepsilon -M}^{\varepsilon }$*.*
 ***9.***
${\left\{{\hat{O}}_{ij}(k)\right\}}_{k=\varepsilon -M}^{\varepsilon }\to {\hat{h}}_{i}^{j}\left(t\right)$*.*
 ***10.***
${\hat{h}}_{i}^{r}\left(t\right)$
* = *
${\hat{h}}_{j}^{r}\left(t\right)+{\hat{h}}_{i}^{j}\left(t\right)$
*,*
ε=1*.*
 ***11.***
*broadcast*
${\hat{h}}_{i}^{r}\left(t\right)$*, correct clock speed.*
 ***12.***
*quit.*


The term r provides the global reference clock. Both the clock offset correction and clock skew compensation of the time synchronization algorithm are used to force the logic time between node i and r to be consistent. In the initialization period of multiple TTMEs, ε is defined as a counter to record the number for clock offset estimation ${\hat{O}}_{ij}$ (Algorithm 2, line 1). The constant M is employed to limit the number of MLE observations (Algorithm 2, line 2). ${\hat{h}}_{j}^{r}\left(t\right)$ is the relative clock speed offset estimation for j. Once node j estimates the ${\hat{h}}_{j}^{r}\left(t\right)$ successfully, it broadcasts a time synchronization packet which is filled with ${\hat{h}}_{j}^{r}\left(t\right)$ immediately (Algorithm 2, line 3). The clock offset MLE is initialized once multiple TTMEs are finished. The clock offset estimations ${\hat{O}}_{ij}(\varepsilon )$ are recorded as the set of observations for clock speed offset estimate (Algorithm 2, line 4–6). The clock speed offset $h_{i}^{j}\left(t\right)$ is calculated once the set of ${\hat{O}}_{ij}$ is big enough (Algorithm 2, line 7–9). The relative clock speed offset ${\hat{h}}_{i}^{r}\left(t\right)$ is the sum of ${\hat{h}}_{i}^{j}\left(t\right)$ and ${\hat{h}}_{j}^{r}\left(t\right)$ (Algorithm 2, line 10). Node i employs ${\hat{h}}_{i}^{r}\left(t\right)$ to compensate its local clock speed to root r, at the same time the ${\hat{h}}_{i}^{r}\left(t\right)$ is broadcasted to help its son nodes to correct clock speed offset (Algorithm 2, line 11).

### 5.1. MLE Clock Offset Estimation

**Figure 5:**
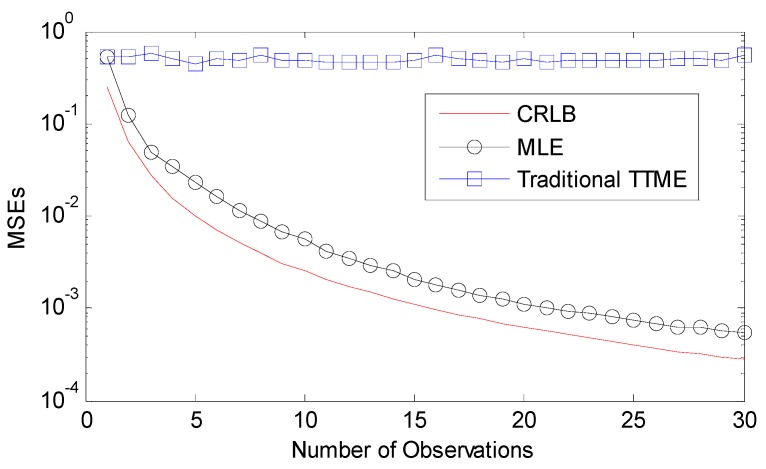
CRLB and MSE of clock offset estimation, assuming the variable delay is an exponentially distributed random variable with mean α=1.

The MLE for clock offset with unknown fixed delay D is proven by Jeske [38]. The random variable delay $d_{x}$ and $d_{y}$ are described as an exponential distribution. The CRLBs of MLE is proven by Noh et al. [40]. The clock offset MLE is given by [38]:

(23) $$\hat{O}=\frac{\underset{1\leq k\leq N}{\min}U_{k}-\underset{1\leq k\leq N}{\min}V_{k}}{2}$$

where $\hat{O}$ is the MLE for clock offset. Its CRLBs is given by [40]:

(24) $$Var\left(\hat{O}\right)=\frac{a^{2}}{4N^{2}}$$

where α is defined as the mean of $d_{x}$ and $d_{y}$, N is number of times for TTME, and also is number of observations. The mean square errors (MSEs) of clock offset estimation $\hat{O}$ under exponential distribution are shown in Figure 5.

As the TTME observations increase, the MLE achieves a higher precision for clock offset estimation. The traditional time synchronization algorithms like TPSN yield rough clock offset estimations, while the clock offset estimations error of MLE are reduced as the TTME observations increase. The MSEs of MLE fall faster and closer to the CRLB.

**Figure 6:**
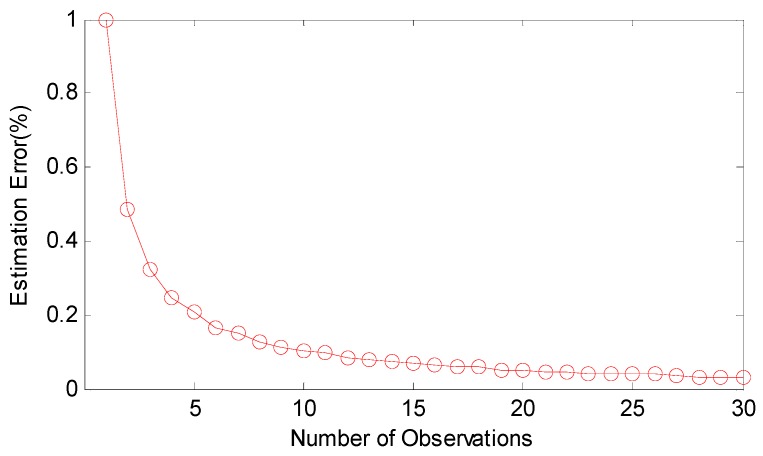
The clock offset estimation improvement to MLE. Compared to the traditional method, the MLE estimation error is reduced fast in the first five observations and slows down after the eighth point.

If $T_{shift}<T_{o}$ there is the largest probability to satisfy the implicit premise that all the observations have the same fixed clock offset, so the number of observation is limited by $\alpha_{ij}$ and the speed of TTME. The proposed multiple TTMEs algorithm maximizes the N by optimizing the TTME. The improvement of multiple TTMEs for clock offset MLE is shown in Figure 6.

### 5.2. Linear Regression Clock Speed Estimation

The clock offset correction is just to force nodes to have a consistent logic time at the correction time. While the h(τ) of nodes based on the clock frequency offset is different, these nodes get different C(t) at the time range t, so the clock offset is continuously changing and clock skew is introduced. Many time algorithms employ resynchronization to restrain the skew but this is not efficient for WNS, so clock speed correction is important and efficient to optimize the clock skew for WSN time synchronization protocols.

We use the latest M observations ${\left\{O_{ij}(k)\right\}}_{k=1}^{M}$ of clock offset estimation to estimate the clock speed. The least squares method is used to regress these observations. The slope of the regression function is the clock speed offset ${\hat{h}}_{i}^{j}\left(t\right)$. There is jitter for the clock frequency and the jitter is caused by the environment changes. We assume that the clock frequency offset and $h_{i}^{j}\left(t\right)$ is fixed for a limited time. Equations (25) and (26) are the models for the clock offset and clock speed:

(25) $${\hat{O}}_{ij}\left(t\right)=h_{i}^{j}\left(t\right)t+O_{ij}(0)$$

(26) $$h_{i}^{j}\left(t\right)=h_{j}\left(t\right)-h_{i}(t)$$

${\hat{O}}_{ij}(t)$ is the MLE for the relative clock offset between node i and j at time t. t is the local time of node i estimating clock offset, i.e., the time stamps ${\left\{T_{k,1}\right\}}_{k=1}^{n}$. j is the reference node, if $h_{j}\left(t\right)$ = 1, $h_{i}\left(t\right)+h_{i}^{j}\left(t\right)=1$. If $h_{i}^{j}\left(t\right)>0$ and the clock speed of node j is faster than i, node i should speed up $h_{i}\left(t\right)$. If $h_{i}^{j}\left(t\right)<0$ and the clock speed of node j is slower than i, node i should slow down $h_{i}\left(t\right)$.$\;h_{i\to j}\left(t\right)$ is speed correction coefficient and the logic clock of i could be rewritten as:

(27) $$L_{i}\left(t\right)=\delta h_{i\to j}\left(t\right)C_{i}\left(t\right)+x_{i}\left(0\right)$$

(28) $$h_{i\to j}\left(t\right)=h_{j}\left(t\right)=h_{i}\left(t\right)+h_{i}^{j}\left(t\right)$$

The clock speed correction coefficient $h_{i\to j}\left(t\right)$ of node i is given by Equation (28). The algorithm employs $h_{i}^{j}\left(t\right)$ to optimize the local clock speed $h_{i}\left(t\right)$, and aims to make node i and j have the same clock speed.

M should be set at an appropriate value. A small M leads to a small subset of clock offset estimate observations for linear regression. The clock offset estimate error of any observation will introduce a larger error into the clock speed offset estimation. Although more clock offset estimate observations lead to a better linear regression, a long time is needed to collect these observations, so it is not efficient. Even worse, if the environment is changing during this period, the relative clock speed is never fixed and continuously changing, so linear regression is unusable.

## 6. Simulation Results and Discussion

A simulation platform based on the True-Time 2.0 toolbox was established to perform the experiment. As we have discussed, TPSN is a typical traditional TTME algorithm, we compared the performances of our algorithm with TPSN. We set the hardware clock frequency as 32.768 kHz, $a_{max}=40$ ppm the clock drift as 0.2 ppm. For both traditional TTME in TPSN and the multiple TTMEs the statistical properties were compared.

The line model with five nodes was established. The nodes identification numbers were set as 1 to 15. The networks topology was set as line 8→7→6→5→4→3→2→1←9←10←11←12←13←14←15. Node 1 was root and in the middle of bridge. ρ=0.1, N=15, M=9. The interval of re-synchronization was 20 s. Both the interrupt handling delays of sender and receiver were random variables value with an exponential distribution, λ=150 μs.

### 6.1. The Synchronization Error

The accuracy clock offset estimation is the important factor for both setting up time synchronization and holding that time synchronization. The time synchronization is set up by correction of ${\hat{O}}_{ij}(t)$ and ${\hat{h}}_{ij}\left(t\right)$ of j. The accurate clock offset estimations are important for an efficient bridge monitoring WSN time synchronization algorithm.

**Figure 7:**
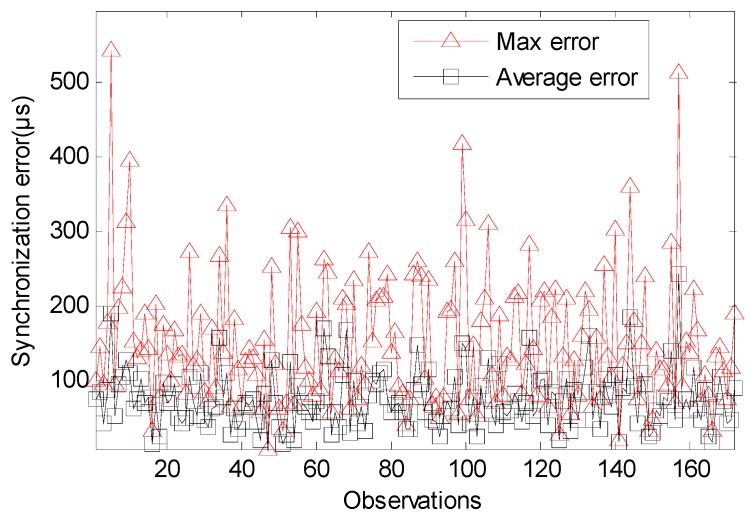
The absolute synchronization error of traditional TTME.

**Figure 8:**
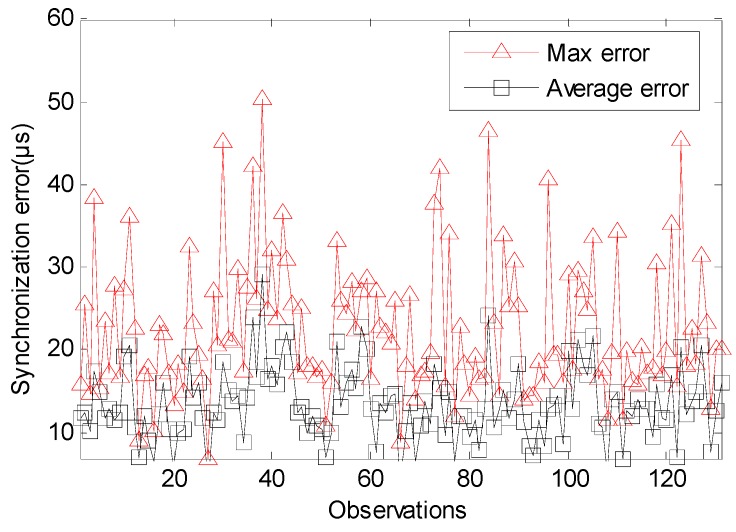
The absolute synchronization error of multiple TTMEs.

For arbitrary i and j ($j\in N_{i}$), the synchronization error $error_{ij}\left(t\right)={\hat{O}}_{ij}\left(t\right)-O_{ij}\left(t\right)$. Its max estimate error $error_{max}\left(t\right)=max(error_{ij}\left(t\right))$. The average estimate error is $error_{average}\left(t\right)={\left\{\bar{error_{ij}}\left(t\right)\right\}}_{i\in V,j\in N_{i}}$. Figure 7 show that the max error of traditional TTME is 542 μs. Figure 8 show that the max error of multiple TTMEs algorithm is 50 μs, we set a small value for $T_{wait}$ so that the max estimate error caused buy clock drift is a single $T_{tick}$, i.e., ${\hat{O}}_{ij}\left(t\right)\in \left\{0,\;30.518\right\}\;\mu s$, while the time synchronization error is mainly caused by $d_{x}$ and $d_{y}$. The multiple TTMEs for MLE clock offset estimate reduce the error efficiently.

The average error of traditional TTME is about 75 μs, the standard deviation of its estimation error is 74 μs. The average error of multiple TTMEs algorithm is about 13 μs, the standard deviation of its estimation error is 8 μs. The other statistical property shows in Table 1. The probability for the multiple TTMEs algorithm that the time synchronization error is smaller than $T_{tick}$ is 95 percent, while the probability for traditional TTME is 32 percent, so the multiple TTMEs algorithm is better to restrict the estimate errors caused by variable delays.

**Figure 9:**
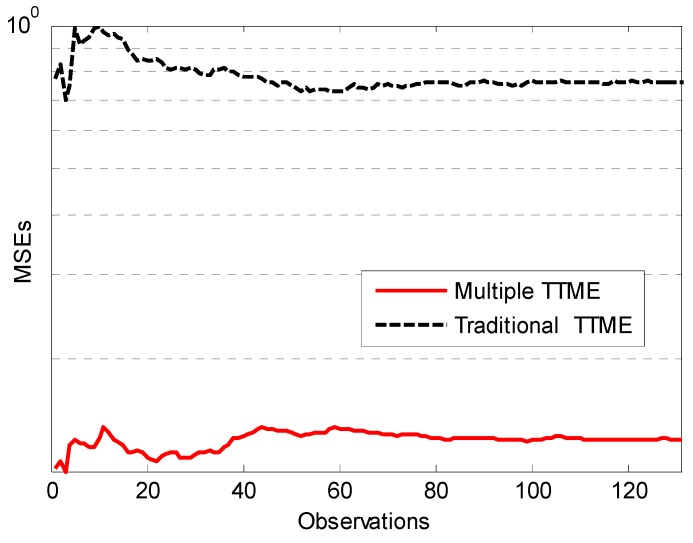
The normalization MSE of the average synchronization error.

Since the ideal clock source granularity $T_{tick}=30.518\;\mu s$ (clock source period), as discussed in Equations (12) and (13), it’s reasonable that if the hardware clock offset is less than one $T_{tick}$ it is difficult to estimate this clock offset. Figure 9 shows the MSE of an average clock offset estimation. Because of the increasing number of observations, the MSE is significantly smooth. It shows that the multiple TTMEs algorithm is better than traditional TTME.

### 6.2. The Clock Skew

The clock offset estimate error is always introduced to the clock speed estimation and manifests as clock skew. To restrain the clock skew could improve the precision of synchronization and extend its hold time. To get a long time synchronization without frequently resynchronizing, it’s an efficient way to employ an accurate clock speed estimation to restrain the clock skew.

**Figure 10:**
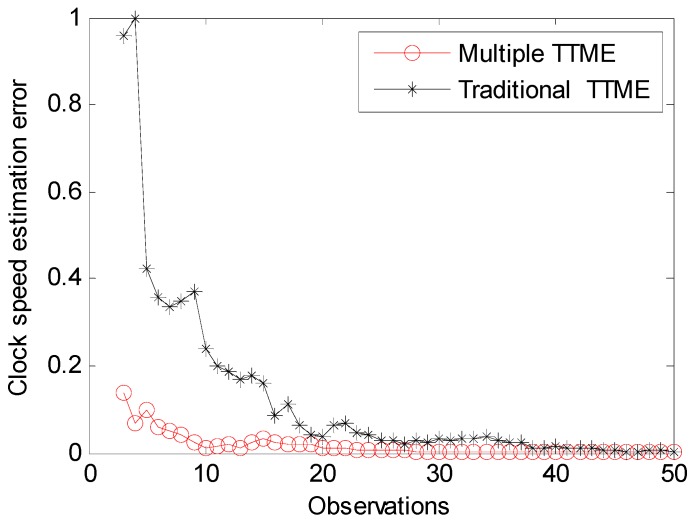
Clock offset estimate precision and clock speed regress precision.

The multiple TTMEs algorithm provides a more accurate clock offset estimate and the clock skew estimation error needs to be discussed. We have simulated the clock speed estimate efficiency of traditional TTME and multiple TTMEs. Figure 10 shows the convergence rate. The multiple TTMEs algorithm is faster and smoother. The multiple TTMEs algorithm needs fewer observations to obtain a higher precision clock speed estimation, it has more advantages for the WSN time synchronization applications with poor storage and calculation ability.

**Figure 11:**
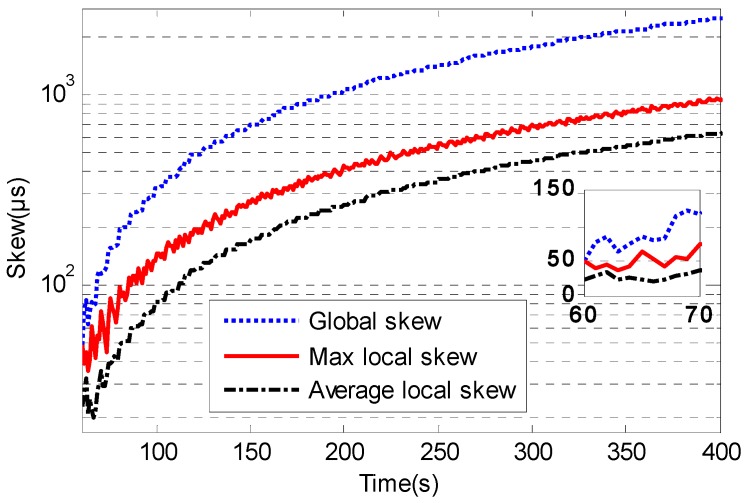
The skew of traditional TTME.

**Figure 12:**
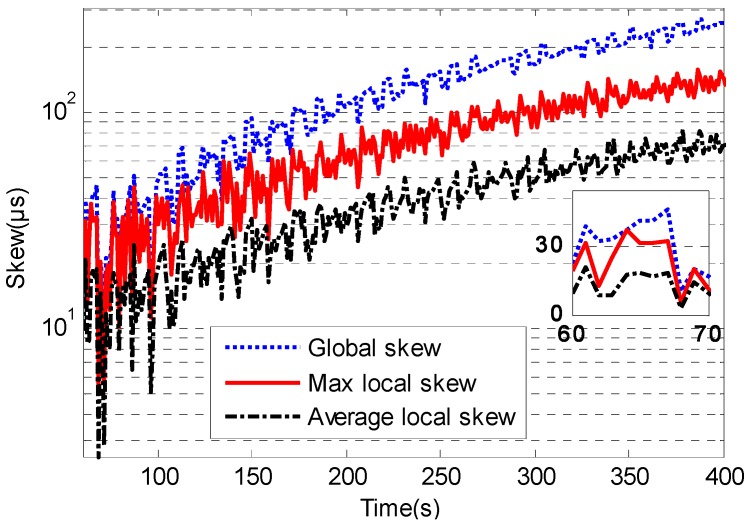
The skew of multiple TTMEs.

The clock skew could be reduced visibly when the time synchronization algorithm employs clock speed compensation. Figure 11 and Figure 12 show the local clock skew and global skew for traditional TTME and multiple TTMEs. The first 50 s is the initialization period for time synchronization. The accurate ${\hat{h}}_{i}\left(t\right)$ estimation is due to the high precision clock offset estimation and it will minimize the clock skew. The local skew of multiple TTMEs algorithm is better than traditional TTME. The skew of the multiple TTMEs algorithm is smaller than that of traditional TTME.

As the local skew increases, the global skew will inevitably increase. Since the max frequency offset is 40 ppm, if the time synchronization algorithm has no clock skew estimation, the global skew increasing rate is up 2400 μs for every 60 s by Equation (11).

The global skew of traditional TTME increases at the speed nearly 438 μs for every 60 s. The average speed of local skew increase for traditional TTME is nearly 108 μs for every 60 s, and the max speed is near 160 μs for every 60 s.

The global skew of the multiple TTMEs algorithm increases at a slower rate, as the speed is nearly 47 μs for every 60 s. The speed of increase of the local skew for the multiple TTMEs algorithm is lower also, as the average value is near 14 μs for every 60 s, and the max value is nearly 27 μs for every 60 s.

## 7. Conclusions

This paper presents an easy method for MLE time synchronization for bridge monitoring wireless sensor networks. Various sensors are employed for sensing the real-time information of a bridge and these sensors are deployed on the bridge in the wireless bridge monitoring system, so a same time notion is important for multiple sensor data fusion. Time synchronization algorithms aim to build a consistent time notion. The proposed time synchronization algorithm promises an effective observations set for MLE. We have discussed the error sources of TTME clock offset estimation and details of different clock offset models. Based on the important assumption that there is fixed offset for TTMEs, a multiple TTMEs mechanism is proposed to guarantee this assumption. With multiple TTMEs, an observation set which has fixed offset can be created to meet the clock offset MLE. The simulation results show that compared to traditional TTME algorithms like TPSN, the multiple TTMEs algorithm can achieve a higher time synchronization precision. The algorithm uses a hierarchical topology spanning tree to build time synchronization quickly in the networks. As Tomonori, and Spencer [1] discussed, it is acceptable for SHM that the time synchronization error be smaller than a millisecond, so the proposed approach in this paper is suitable for bridge SHM.

Further evaluation of our protocol is needed. While there is large random latency in the node response process, the communication cost of multiple TTME retries should be considered. The timeout parameter ρ should be optimized to make the time synchronization algorithm converge fast.

## Figures and Tables

**Table 1:** The synchronization error probability.

	Probability (%)	
	<Average Error	<$T_{tick}$
**Traditional TTME**	62 (75 μs)	32 (30 μs)
**Multiple TTMEs**	51 (13 μs)	95 (30 μs)
